# The Gut–brain–adipose Axis in Ultra-processed Food and Obesity: A Mechanistic Synthesis and Its Implications for Food Classification

**DOI:** 10.1007/s13679-026-00745-0

**Published:** 2026-07-29

**Authors:** Jimmy Chun Yu Louie

**Affiliations:** Discipline of Dietetics, Swinburne University of Technology, Level 2, Swinburne Place West Building, 1 John St, Hawthorn, Melbourne, VIC 3122 Australia

**Keywords:** Ultra-processed food, Nova classification, Gut microbiome, Hypothalamic inflammation, Adipose tissue, Metabolic endotoxaemia, Obesity

## Abstract

**Purpose of Review:**

Consumption of ultra-processed food (UPF) tracks closely with obesity across populations, and the Nova classification has become the dominant tool for capturing that exposure. Why UPF promotes weight gain is a separate question, and the biological answer has accumulated in fragments. I draw those strands together and ask whether three mechanisms usually studied in isolation: gut microbial disruption, hypothalamic inflammation, and adipose tissue dysfunction, are better understood as one connected system, and what that would mean for how UPF is classified.

**Recent Findings:**

Three experimental literatures have converged on a shared pathway. Dietary emulsifiers and non-sugar sweeteners alter microbial composition and weaken the intestinal barrier, raising circulating lipopolysaccharide. In animal models this signal reaches the hypothalamus, where it activates inflammatory pathways, recruits glia, and blunts the leptin response that normally limits intake; though whether the same sequence operates in humans remains unestablished. Visceral fat that expands under this regime secretes its own inflammatory load, which feeds back onto both the gut and the brain. A 2025 UK Biobank analysis added a human imaging dimension, reporting structural differences in feeding-related brain regions that scaled with UPF intake and were only partly explained by adiposity.

**Summary:**

The evidence coheres best when the three arms are read as a single self-reinforcing loop in which each influences the others. Within that frame, the limitation of the Nova classification becomes specific and tractable: Group 4 mixes products that engage the loop strongly with products that barely touch it. This review sets out where the mechanistic evidence is firm, where it remains thin, and how an attribute-aware refinement of Group 4 might be tested.

## Introduction

Obesity prevalence has roughly doubled worldwide since 1990 [1], and conventional dietary advice built around individual nutrients has not reversed the trend [2, 3]. The composition of the food supply shifted over a longer horizon. Across the second half of the twentieth century, and accelerating from the 1990 s, industrially formulated products high in additives and low in intact plant structure progressively displaced home-prepared meals, first in high-income countries and more recently in low- and middle-income settings [4, 5].

**Fig. 1:**
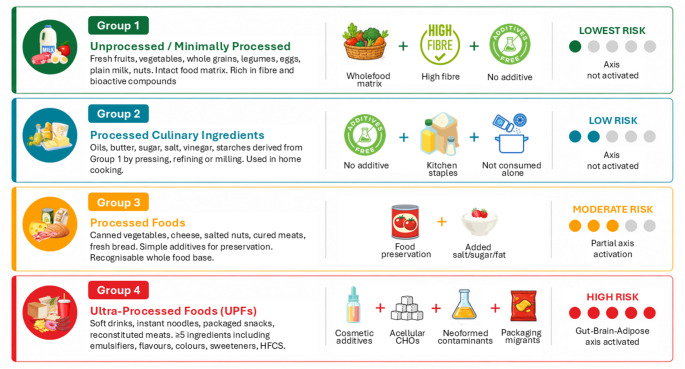
Nova food classification system and purported obesity risk gradient

The Nova system sorts foods by the degree and purpose of processing rather than by nutrient content [6, 7] (Fig. 1). Its fourth group, ultra-processed food (UPF), gathers products defined by industrial formulation and by ingredients not commonly found in domestic kitchens, among them emulsifiers, modified starches, non-sugar sweeteners, colours, and flavour compounds [6]. The epidemiology connecting this group to obesity is now extensive. A 2024 umbrella review covering 45 meta-analyses and close to ten million participants graded the association with adiposity as highly suggestive [8]. The association is uneven across the intake range. In the EPIC cohort, elevated type 2 diabetes risk was confined to the highest consumers, with a hazard ratio above 1.0 only in the top quartile, corresponding to intake above 40% of energy, across all adjustment models [9]. Threshold behaviour of this kind is difficult to reconcile with treating Group 4 as a single homogeneous exposure.

Evidence from randomized trials also started to emerge. A two-week inpatient trial reported that an ultra-processed diet drove an extra 508 kcal per day and measurable weight gain against a minimally processed comparator matched for presented sugar, fat, fibre, and sodium [10]. What that trial could not isolate is mechanism. The diets differed in many ways at once, so the excess intake cannot be assigned to any single feature of processing [11, 12]. A subsequent crossover trial linked UPF-related weight gain partly to faster eating and reduced chewing, pointing to texture and eating rate as intake-relevant properties that vary with processing [13].

Three lines of mechanistic work have grown up around the problem. One concerns the gut microbiome and the intestinal barrier [14, 15]. A second concerns inflammation in the hypothalamic circuits that govern appetite [16–18]. A third concerns the adipose tissue that stores the resulting surplus and behaves as an endocrine organ [19–21]. These literatures have largely developed on separate tracks, with separate model systems and separate journals [4].

Running alongside the biology is an unresolved debate about the classification itself. Critics note that Group 4 places ultra-processed wholegrain bread beside cola, that it quantifies neither additives nor processing intensity, and that coding the same products differently across studies shifts prevalence estimates by double-digit margins [22–24]. The World Health Organization has recently initiated formal guideline development on UPF [25], signalling that the tension between epidemiological utility and mechanistic specificity is now being addressed at the policy level.

This review pursues two linked questions. First*, do the three mechanistic arms describe one system or three?* Second, if they describe one system, *what does its structure imply for the way ultra-processed food is classified and acted upon?* To address these questions, I weigh the evidence for each arm, examine how the arms connect, and consider how a mechanistically informed refinement of the classification might be framed and tested.

The system I describe is a heuristic for connecting processing-specific exposures to downstream physiology. It is not an exhaustive account of energy homeostasis, which integrates ghrelin, GLP-1, insulin, glucagon and other signals across the central nervous system, sympathetic outflow, liver, skeletal muscle and adipose tissue. My aim is narrower: to trace how specific attributes of Nova Group 4 foods engage a subset of these pathways in a mutually reinforcing way.

## The Nova Classification and Its Open Questions

### Epidemiological Reach

Nova rests on a single idea: industrial processing can change how a food behaves in the body through additives, neoformed compounds, and the disruption of intact food structure, independently of the macronutrient label on the package [5–7]. Monteiro's early Brazilian data showed that the share of UPF in the diet captured population-level shifts in diet quality and obesity more effectively than nutrient-based approaches [26, 27]. Large cohorts since then, among them NutriNet-Santé [28], UK Biobank [29], and NHANES [30], have repeated the finding for BMI, waist circumference, and visceral fat. Meta-analyses of approximately thirteen prospective cohorts consistently show higher risks of overweight and obesity with greater UPF intake, with dose–response analyses indicating around 10% increases in risk per 10% higher intake in large cohorts such as NutriNet‑Santé [31, 32]. Few diet–disease associations in nutritional epidemiology have been replicated as consistently across populations, cohorts, and analytic approaches.

### Where the Category Strains

The difficulty is internal heterogeneity. Group 4 treats a packaged wholegrain loaf and a sugar-sweetened soft drink as equivalent [12, 33], yet their associations with cardiometabolic risk diverge sharply. Several cohorts find that ultra-processed breads, plain yoghurts, and some dairy desserts sit at or below neutral for risk, while processed meats, soft drinks, and packaged snacks carry most of the adverse signal [9, 34–36]. A classification that cannot separate these within a single group offers limited traction at the bedside.

A second problem is granularity. Nova records neither which additives a product contains nor how much [37, 38]. Carboxymethylcellulose (CMC) and polysorbate 80 (P80) disturb the intestinal barrier in animal and ex vivo human work [15], yet most Group 4 products contain neither, and Nova cannot tell the two situations apart [38]. Reliability compounds the issue: applying different coding conventions to the same foods has shifted estimated UPF prevalence by as much as fifteen percentage points in one population [39]. None of this overturns the epidemiology. It does mark out where a mechanistic account could sharpen a tool that currently works at the level of the whole diet but blurs at the level of the individual product [12, 40, 41].

## First Arm: Disruption of the Gut Microbiome and Barrier

### Food-level Factors and the Gap to Biology

Before turning to individual components, one caveat frames the section. Food matrix integrity, energy density and texture vary systematically across processing levels and shape intake through routes that are only partly mapped onto the biological arms described below [42]. The bridge between these food-level properties and downstream physiology remains incompletely characterised, and I return to it in the research agenda.

### Components that Perturb the Gut

A diverse microbiome with active short-chain fatty acid production supports barrier integrity and metabolic health [43–46]. Several features of UPF have been proposed to erode that state, through routes that differ in how directly they have been demonstrated.

Emulsifiers are the clearest example. In mice, dietary CMC or P80, both common in ice cream, dressings, and reconstituted meats, thinned the protective mucus layer and allowed bacteria to encroach on the epithelium, with low-grade inflammation and metabolic syndrome following [15]. Transferring microbiota from emulsifier-fed animals into germ-free recipients carried the adiposity phenotype with it, which points to the microbiome as a mediator rather than a bystander [15]. These animal exposures used concentrations above typical human intake [47], a point I return to when grading the evidence. An ex vivo human study subsequently demonstrated that these emulsifiers directly alter human microbiota composition and gene expression, potentiating intestinal inflammation [48]. The 2022 human randomised controlled feeding trial of CMC confirmed detrimental effects on microbiota composition and metabolome in healthy adults [49], and a recent placebo-controlled randomised trial testing five emulsifiers in people with Crohn's disease demonstrated measurable effects on gut inflammation, permeability, and microbial composition at doses relevant to typical dietary exposure [50]. In the NutriNet-Santé cohort, emulsifier intake from industrial food products tracked with markers of gut barrier disturbance and cardiovascular risk [51–53]. The human signal is not uniform, however. In the trial by Wellens [50], not all emulsifiers produced detectable effects, and commonly used agents such as soy lecithin showed little impact on inflammation or barrier function [54], which cautions against treating the emulsifier class as biologically homogeneous.

Non-sugar sweeteners act along a parallel path. Saccharin impairs glucose handling by reshaping the microbiome, an effect abolished when the microbiota are cleared with antibiotics [55]. Experimental studies further show that saccharin and sucralose lower *Lactobacillus* and *Bifidobacterium* populations and reduce faecal short-chain fatty acid (SCFA) concentrations [55, 56]. More recent work has extended this picture. A 2022 randomised trial showed that responses to non-nutritive sweeteners are personalised through individual microbiome configurations, reshaping community structure and downstream glucose handling in a manner that varies from person to person and complicates any single population-level estimate [56]. Syntheses have since linked these shifts to immunometabolic signalling [57]. This picture is contested. Other randomised trials report negligible glycaemic or microbiome effects at typical intakes [58], and substitution studies find that replacing sugar with non-sugar sweeteners can support modest weight loss [59, 60]. The 2023 WHO guidance reflected this uncertainty, recommending against sweeteners for weight control while acknowledging the evidence base as low in certainty [61].

Acellular carbohydrates act through what they fail to deliver to the colon. Refined starch and high-fructose corn syrup, stripped of the plant cell walls that normally shield carbohydrate from rapid absorption, are taken up high in the gut and deliver little fermentable substrate to the colon [45]. A crossover RCT demonstrated that switching from unrefined to refined carbohydrate diets shifted gut taxa and reduced microbial metabolite diversity [62]. This is mechanistically explained by the loss of microbiota-accessible carbohydrates (MACs): without intact plant cell-wall polysaccharides, fermentative populations dependent on polysaccharide breakdown decline, SCFA production falls, and colonocyte energy supply is compromised through *GPR41*, *GPR43*, and *GPR109A* signalling pathways [63, 64].

Neoformed compounds such as acrylamide and advanced glycation end-products (AGEs), generated during Maillard reactions at high processing temperature, exert genotoxic and pro-inflammatory effects on colonocyte and microbial communities in experimental systems [65]. Evidence for direct gut microbiome perturbation remains limited at regulatory-relevant doses, and this gap is acknowledged in Sect. 6. Packaging migrants including bisphenol-A (BPA) and phthalates, leaching from food contact materials into UPF products, act as endocrine disruptors; recent analyses of plastic food packaging from several countries have documented contamination with endocrine- and metabolism-disrupting chemicals; in experimental systems, such compounds can alter microbial composition and promote adipocyte differentiation [66–68].

### From Barrier Failure to Systemic Signal

These perturbations converge on a leakier intestinal barrier. Tight-junction proteins fall, and LPS from gram-negative bacteria crosses into the circulation [46, 69]. A high-fat, ultra-processed meal can raise circulating LPS two- to threefold above fasting levels [70]. Once in the blood, LPS engages Toll-like receptor 4 across immune cells, adipocytes, hepatocytes, and the microglia of the hypothalamus [46], lighting the inflammatory fuse for the second arm.

## Second Arm: Central Inflammation, Leptin Resistance and Reward Dysregulation

### The Arcuate Nucleus As Appetite Gatekeeper

Appetite regulation is distributed across several brain regions, including the nucleus of the solitary tract, hippocampus and mesolimbic reward areas. I concentrate on the arcuate nucleus of the hypothalamus because the metabolic-endotoxaemia signal has been most directly characterised there. Within it, neuropeptide Y/agouti-related peptide (NPY/AgRP) neurons that promote feeding are balanced against pro-opiomelanocortin (POMC) neurons that promote satiety, integrating leptin, insulin, ghrelin and gut peptides [71, 72]. Inflammation in this region, marked by reactive glia and local cytokine release, uncouples that balance and produces leptin resistance even when circulating leptin is high [18]. Human relevance is anchored by post-mortem evidence of gliosis in the mediobasal hypothalamus of people with obesity [18, 73].

Rodent work fills in the sequence. In rodents, diets rich in processed ingredients trigger hypothalamic inflammation within roughly three days, before body fat has meaningfully increased, which places inflammation early in the sequence [74]. In rodent models, lipopolysaccharide (LPS) acting through Toll-like receptor 4 (TLR4) on microglia activates nuclear factor kappa-B (NF-κB) and induces suppressor of cytokine signalling 3 (SOCS3), an intracellular feedback protein that dampens leptin-receptor signalling through the JAK2/STAT3 pathway [75]. The corresponding sequence in humans is inferred from these models and from post-mortem and imaging data rather than demonstrated directly.

### Imaging the Circuit in People

Human data took a step forward in 2025. A UK Biobank analysis of more than thirty thousand participants used MRI-derived measures to relate brain microstructure to UPF intake [76]. Higher intake was associated with imaging signatures of altered cellularity in the hypothalamus and microstructural differences in reward-related regions including the nucleus accumbens, putamen, and pallidum. Mediation analyses indicated that adiposity and dyslipidaemia explained only part of these associations, implying that ultra-processed foods may influence the brain through pathways beyond simple weight gain [76]. The design is cross-sectional and cannot establish the direction of causation. An alternative reading runs the other way. Established obesity can itself induce hypothalamic gliosis, so the cross-sectional association is equally consistent with adiposity driving the brain changes rather than the reverse. Disentangling the two will require longitudinal imaging across dietary transitions [73].

### Reward as Well As Homeostasis

UPF also acts on reward circuitry. Engineered combinations of fat, sugar, salt, and glutamate push dopaminergic activity in the accumbens and orbitofrontal cortex past the levels that ordinary food evokes [77]. In rodents, sustained exposure lowers dopamine D2 receptor availability and dampens dopaminergic tone, producing compulsive intake patterns that resemble addiction [78]. The nucleus accumbens findings from UK Biobank imaging study are consistent with reward-circuit remodelling in humans [76]. This hedonic dimension may help explain why advice to eat less alone is often insufficient.

## Third Arm: Adipose Tissue Dysfunction and the Closing of the Loop

### Visceral Fat As an Inflammatory Organ

Adipose tissue secretes hormones, cytokines, and lipid mediators that set whole-body insulin sensitivity and immune tone [79]. Visceral fat, which expands readily on energy-dense low-fibre food, becomes hypertrophic, recruits macrophages that surround dying adipocytes in characteristic crown-like structures, and shifts its secretory profile towards inflammation, with leptin and tumour necrosis factor-alpha (TNF-α) rising as adiponectin falls [21, 80–82]. In the PREDIMED-Plus imaging sub-study, a higher proportion of energy from UPF predicted greater visceral fat and total fat mass at one year after adjustment for total energy intake and Mediterranean-diet adherence, a residual association consistent with processing-specific effects on fat biology [83].

*Trans*-fatty acids, generated by partial hydrogenation, illustrate a further processing-specific route, though their contemporary relevance is qualified. Industrial *trans* fats have been restricted in the United States, the United Kingdom and through the WHO REPLACE programme [84], and their contribution from UPF has fallen substantially where enforcement is effective. Residual exposure persists through incomplete regulation in some markets, ruminant *trans* fats and repeated high-temperature frying. Adverse cardiometabolic effects are reported above roughly 1% of energy [85], and saturated fatty acids, quantitatively more prominent in many UPF-rich diets, warrant parallel consideration [86]. Beyond their well-established pro-inflammatory effects mediated through NF-κB and NLRP3 inflammasome activation in adipose macrophages, they promote hepatic steatosis and insulin resistance through mechanisms partially independent of energy intake [87, 88]. Rodent studies demonstrate that *trans*-fat exposure causes greater hepatic and visceral fat deposition than calorically equivalent saturated fats, consistent with adipose dysfunction beyond energy density [89]. Dysfunctional white adipose tissue contributes to systemic metabolic disease through impaired lipid handling and inflammatory cytokine secretion, across a range of initiating insults [90]. Oxidised and *trans*-fatty acid by-products of frying and extrusion add to the load by activating TLR4 and the NLRP3 inflammasome in adipose macrophages [85, 91].

### Packaging Chemistry and Adipogenesis

Some of the relevant exposure comes from the wrapper rather than the food. Bisphenol-A and phthalates migrate from can linings, plastics, and sachets and activate PPARγ, the transcription factor that licenses adipocyte differentiation and lipid loading [67]. Chemical screening of consumer plastic packaging has confirmed that a broad range of plastic materials used in food contact carry adipogenic activity, with PPARγ agonism detectable from real-world product extracts rather than only from pure compound exposures [92]. Urinary BPA concentrations track with adiposity in population studies, and substitutes including bisphenol-S (BPS) and bisphenol-F (BPF), now widely used since BPA phase-outs in the European Union and Canada, demonstrate similar endocrine-disrupting properties without equivalent regulatory restriction [93]. It is plausible, though not yet demonstrated for UPF-defined diets, that higher UPF consumption entails greater cumulative exposure to these migrants, given the reliance of such products on plastic and lined packaging. If so, Nova would omit an adipogenic exposure pathway that tends to accompany Group 4 while sitting outside its definitional criteria [66, 94].

### Three Arms, One Circuit

**Fig. 2:**
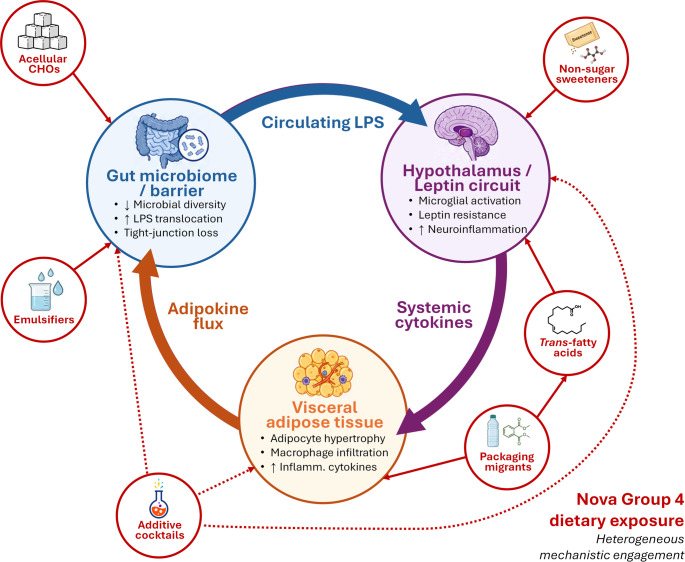
Ultra-processed food driven gut-brain-adipose axis in obesity pathogenesis

Considered together, the three arms behave as an interconnected loop. Chronic leptin elevation from hypertrophic fat may sustain hypothalamic inflammation, plausibly deepening central leptin resistance and helping to keep intake high [95]. Adipose-derived LPS-binding protein and soluble CD14 heighten TLR4 sensitivity in both gut and brain [96]. Gut-derived LPS reaching the portal circulation can activate adipose macrophages directly, at least in animal models [46]. The crosstalk between adipose tissue and the gut microbiome is bidirectional: adipose inflammation alters microbial composition and thermogenic signalling, and microbiome-derived signals in turn regulate adipocyte metabolism and immune infiltration [20]. Each arm can amplify the others. The circuit (Fig. 2) may help explain why UPF-related obesity often responds poorly to interventions aimed at energy intake alone. It is worth being clear about the status of this synthesis. No single study has demonstrated the complete loop operating in humans; the connections are assembled from separate experimental systems, each with its own limitations, and the integrated circuit remains a hypothesis rather than a demonstrated pathway.

## Implications for Classifying UPFs

The mechanistic picture suggests a way to keep what Nova does well while addressing what it does poorly. Nova's processing axis stays. Onto it, I propose adding a second, attribute-based axis that stratifies Group 4 by mechanistic load: the burden of barrier-disrupting additives, the presence of adipogenic exposures such as *trans*-fats and packaging migrants, and the load of rapidly absorbed acellular carbohydrate. A product scoring high on several dimensions would be flagged as mechanistically high-risk; one carrying additives without those features would sit lower. I offer this as a hypothesis for testing; it remains some distance from a usable instrument.

Such a scheme would separate the cases that currently sit awkwardly together, distinguishing a packaged wholegrain loaf with modest additive load from a soft drink that combines acellular sugar, sweeteners, and a bisphenol-lined container. It would also give manufacturers a defined target for reformulation in place of a category they can only enter or leave. A refinement of this kind needs a guard against gaming. A practical difficulty arises here. Many foods contain components with intrinsic emulsifying properties, such as egg yolk or mustard, so a rule cannot simply penalise emulsification as such. The relevant distinction lies between isolated, purified additive emulsifiers used at functional concentrations and emulsification arising from whole-food ingredients. Because a whole-food alternative carries other bioactive components alongside its emulsifying fraction, the burden of proof should rest on demonstrating equivalent or reduced biological effect in human studies rather than on ingredient identity. Without that condition, an attribute axis would invite reformulation that improves the label while leaving the biology untouched.

## Clinical and Public Health Translation

### Targeting the Loop

Viewing obesity through this loop broadens the clinical target from energy balance to the biological circuit that sustains intake. In the one inpatient trial to test this directly, removing UPF lowered intake more than a nutrient-matched comparator over two weeks [10], though the small sample and short duration limit how far the finding can be generalised [11, 97, 98]. The imaging data carry a sobering implication, though: hypothalamic gliosis may not reverse quickly once established [73, 99], so dietary change alone may leave residual leptin resistance, and anti-inflammatory strategies may earn a place in long-term management [73]. In early trials, microbiome-directed approaches such as prebiotic fibre and synbiotics have increased diversity and reduced circulating LPS in some participants [100, 101]. Pairing them with reduced UPF intake has a mechanistic rationale, since continued emulsifier and sweetener exposure would be expected to counteract them, though this combination has not been tested directly.

### Policy Levers

Evaluations of Chile's front-of-pack warning octagons [102, 103] and of the Mexican measures that followed [104–106] report reduced purchases of the targeted products, with the largest shifts among lower-income households. An attribute-aware classification would let such policies aim more precisely: mandatory disclosure of emulsifier identity and dose, ceilings on high-risk emulsifiers in foods marketed to children, limits on bisphenol-lined packaging for acidic products. The 2025 US Dietary Guidelines Advisory Committee folded UPF into its evidence review for the first time, ending a long reluctance to make processing a basis for dietary guidance [107]. A mechanistic account supplies the biological rationale that processing-based policy has so far lacked.

## Limitations and a Research Agenda

Several limits qualify this synthesis. It is a critical narrative review rather than a registered systematic one; my searches of PubMed and Scopus from 2015 to 2026 were structured but did not follow PRISMA. Much of the component-level mechanism comes from animal or in vitro work, with sparse human trial data linking specific attributes to specific arms. The human imaging evidence, though striking, is cross-sectional and silent on direction. The proposed attribute axis is reasoned from mechanism and awaits prospective validation before any regulatory use.

Three substantive objections also warrant statement. Observational UPF associations are confounded by diet quality and socioeconomic position, and adjustment does not fully remove either [41, 108]. The direction of causation between diet, adiposity and hypothalamic change is unresolved. And the human evidence for individual additives remains inconsistent, with positive and *null* trials for the same compounds. These are not fatal to the framework, but they set the terms on which it should be tested.

The same gaps define the agenda. Priorities include: multi-week human trials contrasting emulsifier-containing with emulsifier-free diets matched on other attributes; longitudinal imaging that tracks hypothalamic gliosis as people move between ultra-processed and minimally processed eating; studies of additive mixtures at realistic combined doses; microbiome and metabolomic profiling within the dietary-substitution arms of existing cohorts; and validation of the attribute-stratified scheme against metabolic outcomes in at least two independent populations.

## Conclusion

The available evidence is consistent with a model in which UPF influences obesity through an interconnected circuit. Additives and refined carbohydrates can disturb the gut and weaken its barrier; the resulting LPS may contribute to hypothalamic inflammation and reduced leptin sensitivity; and expanding adipose tissue secretes inflammatory mediators that act back onto gut and brain. The strength of evidence varies along this chain, and several links rest on animal data alone. That layered uncertainty may itself help explain why interventions directed at energy intake alone often achieve limited durable effect. Nova has earned its place by capturing this exposure at the level of the whole diet, and the epidemiology behind it is among the most reproducible in nutrition. Its weakness lies one level down, where Group 4 lumps together products that drive the circuit hard with products that barely engage it. An attribute-aware refinement, stratifying Group 4 by emulsifier load, acellular carbohydrate, neoformed compounds, and packaging chemistry, would carry the mechanistic detail now available into a form clinicians and regulators can use. For clinical practice, the framework suggests attending to this biological circuit alongside energy balance, with sustained reduction of the most strongly implicated UPF products as a reasonable priority pending trial confirmation.

## Key References

- Monteiro, C.A., et al., Ultra-processed foods and human health: the main thesis and the evidence. The Lancet, 2025. 406(10520): p. 2667-2684.
  - *The most comprehensive pro-Nova statement to date — a three-part Lancet Series synthesising the epidemiological case, policy framework, and commercial-determinants argument. Any review engaging Nova's claims must engage this paper directly; it is the primary interlocutor for the field in 2025.*
- Lane, M.M., et al., Ultra-processed food exposure and adverse health outcomes: umbrella review of epidemiological meta-analyses. BMJ, 2024. 384: p. e077310.
  - *Umbrella review of 45 meta-analyses covering ~10 million participants. Provides the strongest epidemiological synthesis available and establishes the graded evidence hierarchy (Class II for obesity/overweight). The foundational citation for stating that UPF–obesity associations are "established."*
- Morys, F., et al., Ultra-processed food consumption affects structural integrity of feeding-related brain regions independent of and via adiposity. npj Metabolic Health and Disease, 2025. 3(1): p. 13.
  - *Large UK Biobank diffusion MRI study (n>30,000) showing that higher UPF intake is associated with structural microarchitectural changes in the hypothalamus, nucleus accumbens, putamen, and pallidum, partially independent of adiposity. The first large-scale human neuroimaging evidence linking UPF to structural feeding-circuit changes. Exceptionally timely and the most novel single citation in the manuscript.*
- Wellens, J., et al., Effect of Five Dietary Emulsifiers on Inflammation, Permeability, and the Gut Microbiome: A Placebo-controlled Randomized Trial. Clin. Gastroenterol. Hepatol., 2026. 24(4): p. 1092-1101.
  - *Placebo-controlled human RCT testing five dietary emulsifiers on gut inflammation, permeability, and microbiome. Among the most recent and methodologically rigorous human trial evidence on emulsifiers; substantially updates the evidentiary status of the gut arm.*
- Juul, F., et al., The role of ultra-processed food in obesity. Nature Reviews Endocrinology, 2025.
  - *Comprehensive narrative review of UPF's role in obesity by a leading pro-Nova team, published in a high-impact endocrinology journal. Provides the most up-to-date statement of the canonical UPF–obesity mechanistic position; an essential reference for situating the current review's contribution.*
- Ludwig, D.S., Is the experimental evidence on ultra-processed food and obesity reliable? Crit. Rev. Food Sci. Nutr., 2026: p. 1-5
  - *Critical appraisal of the experimental evidence on UPF and obesity, including substantive limitations of the Hall et al. RCT. Directly relevant given the review's treatment of causal evidence; from a major collaborator whose methodological position must be engaged.*
- Mauney, E.E., et al., Adipose tissue–gut microbiome crosstalk in inflammation and thermogenesis. Trends Endocrinol. Metab., 2025. 36(8): p. 721-732.
  - *Reviews the adipose tissue–gut microbiome crosstalk in inflammation and thermogenesis. Directly supports the closing-of-the-loop section connecting the adipose and gut arms.*
- Sellem, L., et al., Food additive emulsifiers and risk of cardiovascular disease in the NutriNet-Santé cohort: prospective cohort study. BMJ, 2023. 382: p. e076058.
  - *NutriNet-Santé prospective cohort study specifically on food additive emulsifiers and cardiovascular risk — one of the first large human cohort studies linking specific additive classes (rather than total UPF intake) to disease outcomes. Directly supports the emulsifier-specific evidence strand.*

## Data Availability

No datasets were generated or analysed during the current study.

## Declarations

### Author’s Last Name for PubMed listing

Louie.

### Use of Generative AI

During the preparation of this work the author used Claude sonnet 4.6 (https://claude.ai) in Jun 2026 in order to improve the grammar and clarity of the text he drafted. After using this tool/service, the corresponding author reviewed and edited the content as needed and takes full responsibility for the content of the publication.
